# Nano‐Photonic Crystal D‐Shaped Fiber Devices for Label‐Free Biosensing at the Attomolar Limit of Detection

**DOI:** 10.1002/advs.202310118

**Published:** 2024-07-23

**Authors:** Ignacio Del Villar, Esteban Gonzalez‐Valencia, Norbert Kwietniewski, Dariusz Burnat, Dayron Armas, Emil Pituła, Monika Janik, Ignacio R. Matías, Ambra Giannetti, Pedro Torres, Francesco Chiavaioli, Mateusz Śmietana

**Affiliations:** ^1^ Electrical, Electronic and Communications Engineering Department Public University of Navarre Pamplona 31006 Spain; ^2^ Institute of Smart Cities (ISC) Public University of Navarra Pamplona 31006 Spain; ^3^ Department of Electronic and Telecommunications Engineering Instituto Tecnológico Metropolitano Medellín 050013 Colombia; ^4^ Departamento de Física Universidad Nacional de Colombia – Sede Medellín A.A. 3840 Medellín 050034 Colombia; ^5^ Warsaw University of Technology Institute of Microelectronics and Optoelectronics Warszawa 00‐662 Poland; ^6^ National Research Council of Italy (CNR) Institute of Applied Physics “Nello Carrara” Sesto Fiorentino 50019 Italy; ^7^ Łukasiewicz Research Network – Institute of Microelectronics and Photonics Warszawa 02‐668 Poland

**Keywords:** bloch surface wave, label‐free fiber‐based biosensor, limit of detection, nano‐photonic crystal, thin films

## Abstract

Maintaining both high sensitivity and large figure of merit (FoM) is crucial in regard to the performance of optical devices, particularly when they are intended for use as biosensors with extremely low limit of detection (LoD). Here, a stack of nano‐assembled layers in the form of 1D photonic crystal, deposited on D‐shaped single‐mode fibers, is created to meet these criteria, resulting in the generation of Bloch surface wave resonances. The increase in the contrast between high and low refractive index (RI) nano‐layers, along with the reduction of losses, enables not only to achieve high sensitivity, but also a narrowed resonance bandwidth, leading to a significant enhancement in the FoM. Preliminary testing for bulk RI sensitivity is carried out, and the effect of an additional nano‐layer that mimics a biological layer where binding interactions occur is also considered. Finally, the biosensing capability is assessed by detecting immunoglobulin G in serum at very low concentrations, and a record LoD of 70 aM is achieved. An optical fiber biosensor that is capable of attaining extraordinarily low LoD in the attomolar range is not only a remarkable technical outcome, but can also be envisaged as a powerful tool for early diagnosis of diseases.

## Graphical abstract

An exceptionally low limit of detection is attained using stack of high and low refractive index nano‐layers, instead of single layer approach as for SPR or similar, deposited on D‐shaped single‐mode fibers. This configuration enables to overcome a longstanding trade‐off between sensitivity and figure of merit, thus envisaging optical resonances with narrow bandwidth, while maintaining high sensitivity.

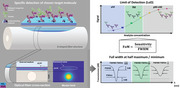

## 1 Introduction

Electromagnetic surface waves (ESWs) are a topic that have been intensively studied for many years, due to their inherent potentialities for application in a broad range of fields of physics, engineering, chemistry, biology, etc. Surface waves typically exist at the interface between two different media, for example either flat or curved stratified structures. Electromagnetic waves can propagate as surface waves if they can be guided along a refractive index (RI) gradient material or along an interface between two media with different dielectric constants.^[^ ^1^ ^]^ In recent years, much ESW‐related research has been motivated by the proliferation of nanotechnology and the growing number of novel (nano)materials that are now available, with unexplored features.^[^ ^2^, ^3^, ^4^ ^]^

ESWs play a key role in the design and development of optical sensing platforms with extreme performance, where the change in the RI of the external medium is a parameter that needs to be assessed precisely and accurately. The optical device is intended to generate an ESW that in turn is linked to an optical resonance. Regardless of the parameter used for optical detection (such as the resonance wavelength, λ_*res*_ [nm], or transmission amplitude, *T*_*min*/*MAX*_ [dB]), any optical resonance exhibits peculiar spectral features that in turn define the performance of the sensing platform.^[^ ^5^ ^]^ There are two main features that dramatically influence the performance of the device: the RI sensitivity, which is inherently related to the type of sensor and sensing mechanism, and the spectral features (i.e., the width and depth/visibility) of the optical resonance, which determine the detection accuracy in the assessment of RI changes. The full width at half maximum (FWHM) is one of the most common parameters used to describe the spectral features of optical resonance.^[^ ^6^, ^7^ ^]^ Consequently, the figure of merit (FoM), commonly defined as the ratio between the sensitivity to RI and the FWHM, is often used as a way to assess the performance of an optical device,^[^ ^8^ ^]^ even if it is not enough alone as much as the sensitivity to RI.

In general, despite the great efforts made by researchers to improve both of the parameters involved in the FoM, there has always been the need for a trade‐off between them: if the RI sensitivity is increased, the FWHM increases, due to a broadening of the optical resonance, mainly caused by higher losses; conversely, if the FWHM is low, the RI sensitivity is relatively low too, due to the small and very confined amount of electromagnetic field interacting with the external medium. This effect has thus far prevented researchers from attaining very low limit of detection (LoD), which are often required for disease‐related applications, and especially for early diagnosis,^[^ ^9^, ^10^ ^]^ when optical sensing platforms are used as label‐free biosensors.

Recently, label‐free optical biosensors based on surface plasmon resonance (SPR),^[^ ^11^, ^12^ ^]^ localized SPR (LSPR),^[^ ^13^ ^]^ interferometry,^[^ ^14^ ^]^ resonating structures^[^ ^15^ ^]^ or other types of optical resonance have been developed, and are now used to a large extent. Of these, optical fiber biosensors have become a hot topic for research due to their excellent performance, as they enable real‐time and direct detection of biological targets with high specificity and sensitivity,^[^ ^16^ ^]^ have small sizes, and permit remote operation and unique light control.^[^ ^17^ ^]^ In addition, the combination of ESWs with fiber devices has attracted strong interest in recent years due to progress in nano‐deposition techniques such as sputtering, dip coating, layer‐by‐layer, and atomic layer deposition, which enable nanometric‐scale films or nano‐structures to be generated on a fiber cladding, a fiber tip, or even inside a fiber when it contains holes.^[^ ^5^, ^18^ ^]^

Lossy mode resonances (LMRs) have also attracted great interest in the domain of optical fiber biosensors during the past few years.^[^ ^16^, ^19^, ^20^ ^]^ As with SPRs, LMRs are generated using thin films with a thickness smaller than the operating wavelength. The real part of the thin‐film permittivity is positive, and is higher in magnitude than both its own imaginary part and that of the material surrounding the thin film.^[^ ^21^ ^]^ The best performance using LMRs has been attained with D‐shaped fibers, where the two polarization states of light (i.e., TE and TM components) can be separated, and there is a single mode in which the light is partly coupled to the thin film, which is separated from the fiber core by a very thin cladding.^[^ ^22^, ^23^ ^]^ This enables the bulk sensitivity to be improved without greatly reducing the FoM, and means that the device can operate in the infrared region with the first LMR, the most sensitive one; this result led to the development of a femtomolar LoD for the detection of immunoglobulin G (IgG) in human serum.^[^ ^24^ ^]^ Moreover, thanks to the help of molecularly imprinted polymers (MIPs), it has been possible to detect cortisol in artificial saliva without the use of a D‐shaped fiber.^[^ ^25^ ^]^

The good performance of LMR in biosensing has led to the exploration, with the same optical structure, of another type of surface wave: Bloch surface waves (BSWs). These are electromagnetic waves that propagate at the interface of two dielectric media, where at least one has a non‐homogeneous periodic structure in the direction normal to the interface.^[^ ^26^ ^]^ BSWs have demonstrated significant potential as biosensing platforms.^[^ ^27^, ^28^, ^29^, ^30^ ^]^ Using a nano‐photonic 1D crystal implemented onto a D‐shaped fiber as the substrate for generating BSWs, a periodic stack of two different dielectric homogeneous layers has been studied by alternating thin layers of SnO_2_ and CuO. This first experiment indicated a moderate RI contrast (0.3 in the NIR^[^ ^31^ ^]^), leading to non‐optimal values for the sensitivity (300–500 nm RIU^−1^) and FWHM (100 nm).

In this study, we propose the deposition of a stack of TiO_2_ and Al_2_O_3_ thin films onto a D‐shaped single‐mode fiber to realize a sensing system with high sensitivity and good FoM at the same time, which enables a very low value of the LoD to be realized. These materials, which have already been applied for optical sensing purposes,^[^ ^32^ ^]^ have a high RI contrast of 0.8, and a low imaginary part, giving rise to the possibility of obtaining optical resonances with a low FWHM^[^ ^20^ ^]^ and hence a high FoM. The proposed optical fiber biosensor, embedded into a microfluidic system and assessed for the detection of a real target analyte (IgG) as one of the gold standards in molecular biosensing,^[^ ^24^, ^33^, ^34^, ^35^, ^36^ ^]^ will not only be capable of attaining outstanding performance with an LoD in the attomolar range, but may will be suitable as a powerful tool for the early diagnosis of several diseases, and particularly neurodegenerative diseases, where the detection of low concentrations of specific biomarkers is urgently needed so that personalized treatments can be started at the onset of the disease.^[^ ^37^ ^]^

## 2 Results

### 2.1 Design of Nano‐Photonic D‐Shaped Fiber Sensors

**Figure 1:**
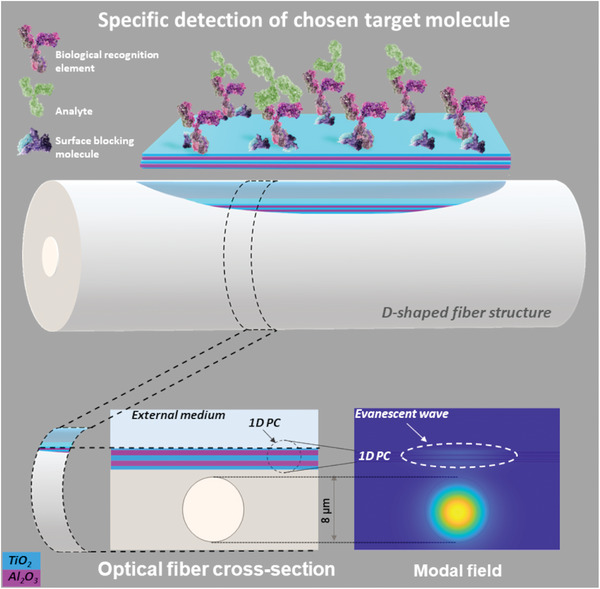
Schematic illustration of a nano‐assembled 1D photonic crystal (1D PC) implemented on a D‐shaped fiber with significantly enhanced figure of merit (FoM) and limit of detection (LoD) at the attomolar level for biomolecule detection.

**Figure** **1** illustrates the concept of a nanomaterial‐embedded optical fiber sensor with enhanced FoM and limit of detection for biosensing. The stack of layers on top of the silica fiber is actually a one‐dimensional photonic bandgap structure (1D PC). The cross section of the D‐shaped fiber shows a five‐layer 1D PC, where the sky blue layers are made of TiO_2_, and the purple layers of Al_2_O_3_.

**Figure 2:**
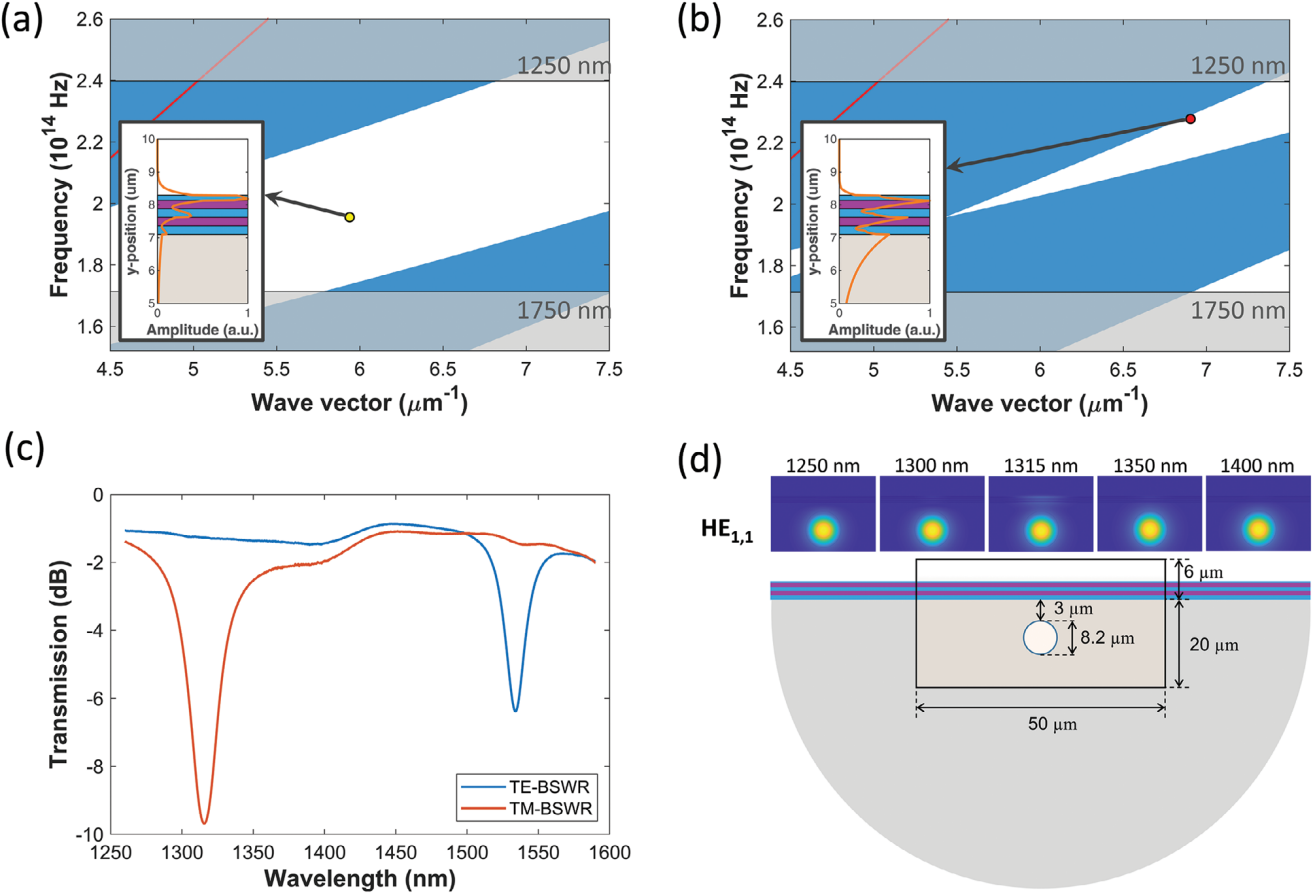
Design and EM analysis of the nano‐photonic crystal D‐shaped fiber structure. Photonic bandgaps for a) TE polarization and b) TM polarization. The yellow and red dots show the BSW resonances when the fiber is immersed in an aqueous medium (n = 1.333), and the insets represent the electric‐field intensity distributions at the 1D PC at the yellow and blue dots. c) Transmission spectra of the D‐shaped fiber with a five‐layer stack, with a last layer of 135 nm thick TiO_2_ and air as the external medium (TE‐BSW shown in blue, TM‐BSW in orange). d) Optical field intensity in the transversal section of the 1D PC‐coated D‐shaped fiber. The simulation window is indicated in the center of the figure. The fundamental mode HE_1,1_ is analyzed for different wavelengths, with a maximum evanescent field at the resonance wavelength.

The first four layers shown in Figure 1 have the same thickness of 260 nm, in order to reduce the degrees of freedom of the design. Combined with a thinner last layer of TiO_2_, this allows the device to generate BSW optical resonances at specific wavelengths λ_*BSW*_ in the operating range of the broadband light source used for the experiments, 1250–1750 nm. The band diagrams for TE and TM polarizations are shown in **Figure** **2**a,b, respectively. In these diagrams, the radiative zones (depicted in blue) and the non‐radiative zones (depicted in white)—also known as forbidden bands—are distinguishable for the propagation within the multilayer stack. The red lines represent light propagation in the surrounding medium (air), and the gray areas represent the wavelengths not covered by the light source. The TE‐BSW is shown as a yellow dot, and the corresponding TM‐BSW as a red dot. It is worth noting that the TM mode lies inside the bandgap region of the developed structure, although it may seem to lie outside or near the bandgap border, as the red dot in the figure has been enlarged to make visualization easier. Figure 2c shows the experimental transmission of the 260/260 nm stack, where the last layer of TiO_2_ (referred to later as Fiber 1) is 135 nm thick and the 1D PC is realized on the flat surface of a D‐shaped fiber (for more details, see Section 4: Experimental Section). The transmission spectra show that when the surrounding medium is air, two resonances are obtained at wavelengths of 1315 nm (corresponding to TM‐BSW) and 1534 nm (corresponding to TE‐BSW). It should be noted that TE and TM resonances have different spectral features (mostly in regard to the depth/visibility of the resonance). The optical field distribution of TM modes has a more significant component in the substrate (i.e., the D‐shaped fiber) than the TE modes, as shown in the insets to Figure 2a,b. The coupling between the core mode and the 1D PC guided mode is therefore easier, with better overlap for the TM modes than for the TE modes. Consequently, the BSW resonances related to the TM modes are deeper and slightly wider than those related to the TE modes, which in turn affects the FoM. However, in terms of performance, we do not expect a large difference, since the ratio between the resonance depth/visibility and bandwidth is very similar for both. In addition, it should be taken into account that the depth/visibility of the resonance tends to decrease when the sensor is inserted into a closed microfluidic system, and it would therefore be preferable to consider the deeper resonance. Figure 2d shows the optical field intensity for the fundamental fiber core mode, HE_1,1_, at the resonance wavelength and in close proximity wavelengths, considering the TM‐BSW, in order to show the enhanced evanescent field at the resonance wavelength compared to the other cases, which determines the presence of a dip centered at this wavelength. Below it, the cross section of the D‐shaped fiber is shown, with the dimensions of the simulation window used to obtain the optical field intensity. This model is used in the following for a numerical analysis of the structure, and more details about it are given in Section 4: Experimental Section.

We note that the thickness of the nano‐photonic crystal layered structure was chosen by calculating the dispersion band diagram for the semi‐infinite multilayer, by means of the transfer matrix method.^[^ ^38^ ^]^ The band diagram of the developed 1D PC for various Al_2_O_3_ and TiO_2_ thicknesses is presented in Table S1 of the Supporting Information. The 260/260 nm stack was chosen both to simplify the construction of the structure by reducing the degrees of freedom in the layer deposition process and to keep the number of layers as low as possible, in order to avoid a time‐consuming fabrication process.

### 2.2 Optical Characterization: Sensitivity, FoM and Other Related Parameters

**Figure 3:**
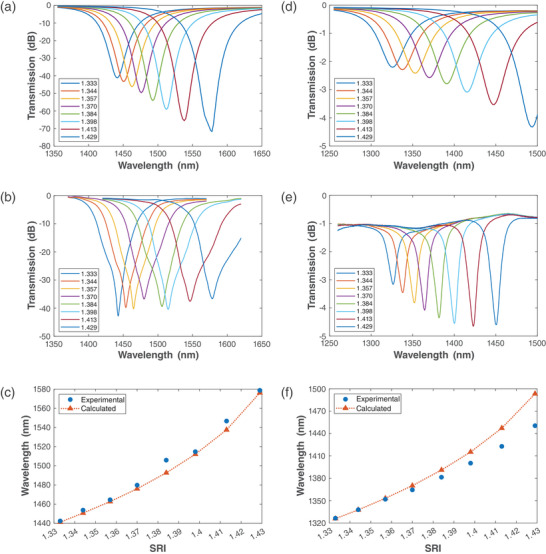
Refractometric response of the proposed structure: a) calculated and b) experimental transmission spectra, and c) resonance wavelength shift for the TM‐BSW; d) calculated and e) experimental transmission spectra, and f) resonance wavelength shift for the TE‐BSW. The last layer in this case was thinner than the last layer used for the TM‐BSW.

When the fiber is covered with water (n = 1.333), the resonance peaks shift to the infrared region. The TE‐BSW cannot be detected, as it is now located outside of the wavelength range monitored by the optical spectrum analyzer, while the TM‐BSW peak is centered at 1442 nm. In order to establish the sensitivity of the structure, Fiber 1 was immersed in various glycerol‐water solutions with different weight percentages (0%, 10%, 20%, 30%, 40%, 50%, 60%, and 70%). **Figure** **3**a,b show the calculated and experimental transmission spectra, respectively, for different aqueous solutions. Figure 3c shows the good agreement between the resonance wavelength shift calculated for the TM‐BSW and the experimental results, with a sensitivity of 1410 nm RIU^–1^ in the refractive index range analyzed here.

To perform RI measurements in aqueous solutions using TE‐BSW, a thinner last layer needs to be deposited, in order to permit the TE resonance in the wavelength range of the optical spectrum analyzer to be visualized. To achieve this, a second D‐shaped fiber was deposited with a five‐layer 1D PC (referred here as Fiber 2). Using the same methodology as for the TM‐BSW, this fiber was immersed in different aqueous glycerol‐water solutions. Figure 3d shows the calculated transmission spectra for different aqueous solutions, while Figure 3e shows the experimental transmission spectra for different aqueous solutions. Finally, Figure 3f shows the agreement between the resonance wavelength shift calculated for the TE‐BSW and the experimental results. The experimental results correspond to a last layer of 87 nm thick TiO_2_, while the numerical results correspond to a layer of 52 nm. From Figure 3, we see that there are some discrepancies between the experimental and simulated results. The simulated results correspond to an analysis of an ideal 260/260 nm 1D PC deposited on the flat section of a D‐shaped fiber; however, as shown in the SEM images (see also the Supporting Information), the deposition process is not exact and does not possess the nanometric accuracy and precision required to ensure the ideal thickness of the thin film, which in practice is expected to vary by up to 10% with respect to the designed thickness. This mostly explains the different outcomes.

Despite the similar wavelengths for both the TE‐ and TM‐BSWs in Figure 2, generated with Fibers 1 and 2, the sensitivity of the TM‐BSW is slightly higher than the sensitivity of the TE‐BSW. This small difference is related to the similarities between these BSWs and LMRs, where the higher sensitivity of TM‐LMRs has been demonstrated.^[^ ^19^ ^]^

The FoM is another important parameter for assessing the performance of optical fiber sensing structures, and can be defined as the ratio between the sensitivity to RI and the full width at half minimum (FWHm) of the resonance.^[^ ^20^, ^39^, ^40^ ^]^ However, other authors have defined the FoM as the sensitivity divided by the full width at half maximum (FWHM),^[^ ^31^, ^41^, ^42^ ^]^ which leads to lower values of the FoM for the structures or to a situation where the FoM cannot be calculated because the resonance exceeds the limit of the optical spectrum analyzer.^[^ ^23^ ^]^ This is not the case here, and the more demanding FWHM is therefore used for the calculations.

A recent paper has also stated that a more realistic definition of the FoM parameter is the ratio between the sensitivity to RI and the FWHM of the resonance, multiplied by the depth of the resonance in dB.^[^ ^25^ ^]^ We therefore consider both this parameter, called the reasonable figure of merit (RFoM), and the traditional FoM in **Table** **1** for the BSWs generated with CuO and SnO_2_ deposited on a D‐shaped fiber,^[^ ^31^ ^]^ for Fibers 1 and 2 (Fiber 3 will be explained later).

**Table 1:** Sensitivity, FWHM and FoM for different BSWs generated with D‐shaped fibers.

PARAMETER	SnO_2_/CuO			TiO_2_/Al_2_O_3_ (five layers)		
	Three layers	Five layers	Six layers	Fiber 1	Fiber 2	Fiber 3
Sensitivity [nm RIU^–1^]	491	317	322	1410	1281	835
FWHM [nm]	146.5	101.5	97.3	90.0	6.0	9.5
FoM (FWHM) [RIU^–1^]	3.35	3.12	3.31	15.7	213.5	87.9
Resonance depth [dB]	15.7	11.3	17	38.3	3.7	4.0
RFoM (FWHM) [RIU^–1^·dB]	52.6	35.2	56.3	599.7	792.1	355.0

According to the simplest definition of FoM, which depends only on FWHM, Fiber 2 has better sensing performance, since the TE‐BSW resonance peaks are narrower. However, if the depth of the resonances is taken into account when calculating the FoM, it can be seen that Fiber 1 has a very similar RFoM, since the TM‐BSW resonances are deeper; this is because they have a more evanescent field around the 1D PC, as shown in Figure 2a,b. This indicates that the performance of TE‐BSW and TM‐BSW is comparable, since depending on the definition of the FoM, each can be seen as better or worse than the other. However, it is clear that in all cases, the results are better in terms of sensitivity and FoM with the structure used in this work, where the main differences are the higher contrast between the high and low RI layers and the reduction in the imaginary part. The performance improvement in terms of sensitivity is around a factor of five, and the improvement in FoM is around 10, compared to previous work with SnO_2_ and CuO layers.^[^ ^31^ ^]^

To evaluate the performance of our structure as a biosensor, Fiber 2 (with the thinner TiO_2_ last layer) was chosen as a substrate to deposit an additional film of Al_2_O_3_, as this material has a RI close to those of biofilms detected by biosensors. This sensor is referred to here as Fiber 3, and a complete analysis of this structure is presented in the Supporting Information (Figures S1–S3, Supporting Information) along with SEM microscope images of the structure (Figure 6a; Figure S4, Supporting Information). The main parameters can also be observed in Table 1, where it is clear that the sensitivity decreases and the FWHM increases, leading to a reduction in the FoM (from 213.5 to 87.9). This effect is reduced slightly if the resonance depth is considered (The increase in RFoM compared to FoM is more significant in Fiber 2 than in Fiber 3). However, in any case, it is clear that the biofilm reduces the FoM by a factor of approximately two. As will be demonstrated later, this effect still permits this structure to obtain very good biosensing performance, mainly due to the fact that the Al_2_O_3_ biolayer is rather thick compared to the thickness of the antibodies and the antigen layer deposited on the D‐shaped fiber.

### 2.3 Optical Characterization: LoD and Other Biosensing Parameters

In this section, we aim to show that the improvements described in the previous section, where the proposed device was used as an optical refractometer, can also be translated into better biosensing performance. This step may seem obvious, but is actually not trivial.^[^ ^43^ ^]^ Demonstrating a significant and noticeable improvement in surface refractive index changes is crucial when utilizing the proposed device as an optical biosensor. To reach this goal, a biosensing experiment was carried out in which we placed the fiber sensors into a custom‐made microfluidic system where the mechanical and thermal effects on fibers were finely controlled, thus enabling long‐term measurements under controlled conditions.

For a clear and direct comparison with state‐of‐the‐art alternatives in the field of optical biosensors based on fibers or other guiding structures, IgG was selected as the target analyte and an antibody‐antigen assay was conducted. To ensure the effectiveness and reliability of the biosensing experiment, each analyte solution containing a different IgG concentration was spiked in human serum as a complex and real biological matrix that contains more than 10000 different kinds of proteins, meaning that this largely mimics clinical settings.^[^ ^44^ ^]^

**Figure 4:**
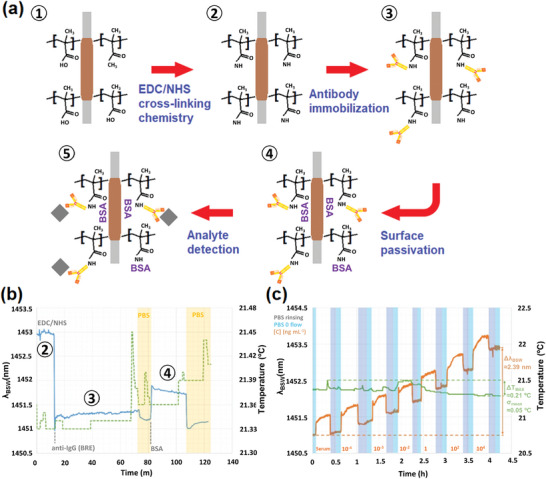
Surface modification processes and related optical responses recorded by the optical fiber device. a) Schematic illustration of all the steps for the implementation of the antibody‐antigen assay: ① deposition of the polymeric functional nano‐layer, ② activation of the functional groups via EDC/NHS cross‐linking chemistry, ③ immobilization of the IgG antibody, ④ blocking of the free functionalities and surface passivation via BSA, ⑤ detection of the target analyte. b) Real‐time response of the optical fiber biosensor at different steps of the functionalization process (blue curve), together with the measured temperature fluctuations (dashed green curve). c) Real‐time response of the optical fiber biosensor at different steps of the detection process (orange curve), together with the measured temperature fluctuations (green curve).

**Figure** **4** illustrates all the steps in the surface modification processes for the 1D structure for the development of the antibody‐antigen assay. A schematic view is shown in Figure 4a. Initially ①, we deposited a nanometric layer of polymer (Eudragit L100 from Evonik), which provided the functional ‐COOH groups. Then ②, these groups were chemically activated via EDC/NHS cross‐linkers, and ③ IgG antibodies were covalently immobilized onto the previously treated region of the fiber. To avoid non‐specific interactions ④, the sensing region was passivated via bovine serum albumin (BSA) blocking agent. Finally ⑤, we detected the anti‐IgG target analyte at different concentrations spiked in 1:10 PBS‐diluted human serum. The dilution of complex biofluids (1:10, 1:100 or even 1:1000), which requires a further preconditioning step when working in real scenarios, is routinely carried out for the assessment of biosensing platforms.^[^ ^45^ ^]^ However, this did not have an impact on the performance of the device in this case, since the analyte concentration, which was spiked in the sample for these measurements, remained unchanged. The details of the protocol are given in Section 4: Experimental Section. Figure 4b shows the details of the real‐time response of the nano‐photonic crystal D‐shaped fiber biosensor at different steps of the functionalization process (shown as the blue curve and text in gray), together with the measured temperature variations (dashed green curve). The overall temperature variation is very small and is in agreement with the level of stabilization of the microfluidic system, and the spikes that are present are related to the change in the flow rate of the injected solution. The levels of the optical signal λ_*BSW*_ before and after the injection of BSA were comparable, meaning that the sensing surface was uniformly and entirely covered. After these steps, the device was ready for the detection process. Figure 4c shows the real‐time response of the nano‐photonic crystal D‐shaped fiber biosensor during the injection of different and increasing concentrations of anti‐IgG (orange curve and text), together with the measured temperature variations (green curve). The analyte concentrations are expressed in ng mL^−1^, ranging from 10^−4^ to 10^4^; the rinsing step in PBS is highlighted with gray windows; and the measuring windows are highlighted in sky blue. During the detection steps, the temperature underwent an overall variation of 0.21 °C with a standard deviation σ of 0.05 °C, and the optical signal λ_*BSW*_ exhibited a red shift of roughly 2.39 nm. We note that the first part of the response (roughly 30 min) corresponds to the specificity test that was carried out using human serum as negative control.

**Figure 5:**
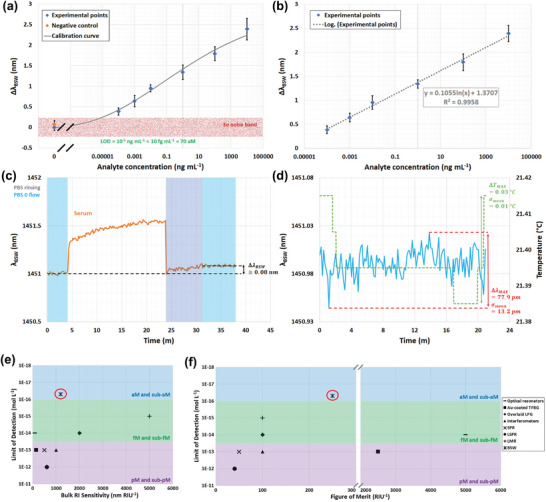
Assessment of the biosensing performance of the nano‐photonic crystal D‐shaped fiber device and performance comparison with state‐of‐the‐art alternatives: a) calibration curve showing the experimental points (blue rhombuses) taken from the measuring windows of the real‐time response (Figure 3c) and related σ, together with a semi‐log fitting function (gray curve). The orange point and related σ refer to the value of the experimental point obtained during the specificity test, while the light red bar represents the 3σ noise band; b) log‐linear plot of the experimental points (blue rhombuses) taken from the measuring windows of the real‐time response (Figure 4c) and related values of σ, together with the regression equation and correlation coefficient; c) details of the specificity test, where human serum was injected as a negative control; d) statistical analysis of the fluctuations in both the optical signal and temperature within a measuring window; performance comparison of optical resonance‐based fiber biosensors over three detection ranges (picomolar shown in violet, femtomolar in green and attomolar in blue): e) bulk RI sensitivity versus LoD, and f) FoM versus LoD. The red circle shows the performance attained with the proposed biosensing platform.

By averaging the values gathered in the measuring windows of Figure 4c, we were able to plot the calibration curve for the proposed nano‐photonic crystal D‐shaped fiber biosensor. **Figure** **5**a shows the calibration curve. The blue rhombuses represent the experimental points, and are shown with their respective values of σ as black error bars. The gray curve represents the sigmoidal fitting function of the experimental points on a semi‐log scale. The logistic function used here is formally equivalent to the Langmuir isotherm, a well‐accepted mathematical model that can be applied to quantify the degree of interaction between ligand binding sites.^[^ ^16^ ^]^ The value of the orange rhombuses and the related values of σ refer to the experimental points obtained during the specificity test. We note that the values are scaled down to zero, which represents the starting baseline achieved during the first measuring window. The light red area represents the 3σ noise band of the blank sample, i.e., the solution at an analyte concentration of zero. In this noise band, an LoD of 10 fg mL^−1^, corresponding to roughly 70 aM, is attained, which to the best of our knowledge is the lowest LoD for an optical fiber biosensor. We also calculated the limit of quantification (LoQ) as an additional and more realistic biosensing parameter, which is related to the 10σ noise band of the blank sample. An LoQ of 9.4 pg mL^−1^ was obtained, corresponding to roughly 63 fM, which represents a breakthrough in the field of optical fiber biosensors. Figure 5b shows the log‐linear plot of the experimental points in Figure 5a, together with the regression equation and the correlation coefficient *R^2^* of 0.9958. To assess the feasibility of the device and its effectiveness as a real biosensor, we tested the specificity in terms of analyte detection by injecting a solution of 1:10 PBS‐diluted human serum as a complex matrix; the aim was to mimic a clinical scenario where a blood sample taken from a patient is allowed to spontaneously clot and then centrifuged. The details of this test are shown in Figure 5c. The change in the optical signal Δλ_*BSW*_ obtained from the measuring windows when the flow stopped was just 0.08 nm before and after the interaction of the functionalized fiber surface with human serum. This value is comparable with the standard deviation of the blank sample, but it is also interesting to note that it is roughly 4.8 times lower than the signal change obtained from the lowest concentration tested, $\Delta \lambda_{BSW}^{100\;fg\;mL^{-1}}$. This illustrates the high degree of specificity of the proposed biophotonic platform.

Further statistical analysis was carried out through a stability test that mimicked a representative measuring window, where the device was surrounded by PBS when the flow stopped. Figure 5d shows the results for a maximum fluctuation of the optical signal $\Delta \lambda_{BSW}^{MAX}$ of 0.078 nm (σ = 0.013 nm) and a maximum variation in the temperature Δ*T^MAX^* of 0.03 °C (σ = 0.01 °C). These data are in agreement with the previous results, and especially with those of the specificity test.

### 2.4 Performance Benchmarking with Other Fiber‐Based Optical Biosensors

To benchmark and validate our work, a complete comparison of the performance of state‐of‐the‐art optical resonance‐based fiber biosensors was undertaken based on the three most crucial parameters: bulk RI sensitivity, FoM and LoD. Of these, the LoD is ultimately the most important quantity. This metric depends on several parameters, such as the inherent sensitivity of the sensor and the width and depth (or visibility) of the optical resonance, which in turn influence the sensor resolution, detection accuracy and FoM, the affinity between the biorecognition element and the target molecule, and thus the mechanical and thermal stability of the system. **Table** **2** summarizes all of this information. It is worth noting that to ensure an effective and accurate comparison in terms of biosensing performance, we considered all of the detection systems that were able to target the same analyte (IgG or anti‐IgG antibodies) in a complex and real environment, i.e., when it was spiked in serum biofluid. In addition, by combining the results gathered in Tables 1 and 2, it is possible to observe that the game changer of this study has been the capability of generating an optical resonance, although with averaged sensitivity to RI, but with low FWHM and hence improved FoM (and RFoM as well), by means of a stack of nano‐materials with high RI contrast and low losses. This has enabled to attain an LoD record of 70 aM for fiber‐based biosensors.

**Table 2:** Performance comparison of different fiber‐based technology platforms for the detection of IgG or anti‐IgG antibody in human serum.

Sensor configuration	Bulk sensitivity [nm RIU^−1^]	FoM [RIU^−1^]	LoD [Molarity]	Reference
LSPR with Au nanorods	753	6.55	0.8 nM	[46]
BIO Bragg gratings on microfibers	–	–	0.66 nM	[47]
LSPR with overlaid Au nanorods	989	6.6	0.6 nM	[48]
LPG combined with MIP	130	16	0.24 nM	[49]
LPG coated with SiO_2_‐TiO_2_ sol‐gel matrix	7075	<100	53 pM	[50]
GO‐coated FBG in combined fiber types	–	–	32 pM	[51]
LMR in multimode fiber coated with ITO	≈4000	≈40	23 pM	[24]
LMR in multimode fiber coated with SnO_2_	5000‐7000	60‐90	6 pM	[24]
LPG at the lowest order cladding mode and near the DTP	8751	–	1.06 pM	[52]
Polymer‐coated etched LPG working in mode transition and near the DTP	–	–	30 fM	[53]
LMR in D‐shaped single‐mode fiber coated with SnO_2_	14 500	≈100	1 fM	[24]
BSW in D‐shaped single‐mode fiber coated with TiO_2_‐ Al_2_O_3_ stack	1281	213.5	70 aM	This work

To complete the performance benchmarking process, several other types of devices were considered, such as TFBGs coated with gold and other 2D nanomaterials,^[^ ^54^ ^]^ interferometers,^[^ ^22^ ^]^ SPR,^[^ ^22^ ^]^ LSPR,^[^ ^36^ ^]^ and optical resonators combined with fiber‐based light‐coupling systems.^[^ ^55^, ^56^, ^57^ ^]^ The performance of each of these types of fiber‐based optical biosensors was compared in terms of the bulk RI sensitivity (Figure 5e) and FoM (Figure 5f) versus LoD. We identified three different LoD ranges: the picomolar (or sub‐pM) level (shown in violet), femtomolar (or sub‐fM) level (shown in green) and the attomolar (or sub‐aM) level (shown in blue). A close look at Table 2 indicates that it is still impossible to realize a device based on optical resonance that possesses both a high RI sensitivity (>1000 nm RIU^−1^) and a high FoM (>1000 RIU^−1^). In practice, devices that have very high bulk RI sensitivity (overlaid LPGs and LMR‐based sensors) are not able to attain a good FoM, due to the intrinsically very large bandwidth of the optical resonance (FWHM, for instance). In contrast, devices that are able to attain a very high FoM (such as TFBGs and optical resonators) do not possess sufficient bulk RI sensitivity. In both cases, the use of nanomaterials to dramatically improve the sensitivity or to functionalize the sensing surface unavoidably introduces some losses, consequently reducing the effective FoM, especially when a biological experiment is carried out. This effect is even more evident when a biological experiment is performed under conditions that mimic a clinical setting where complex and real biofluids are used, such as serum or blood plasma.^[^ ^57^ ^]^ All of these events or conditions have basically prevented for fiber‐based optical biosensors from achieving an LoD in the attomolar range. Conversely, it is possible to observe that the proposed device (shown by the star symbol with a red circle in Figure 5e,f) achieves the best LoD in the literature, to the best of our knowledge, despite the fact that the sensitivity and FoM are not the highest values reported so far. The reason for this stems from the combination of a medium‐high bulk sensitivity with a reasonable value for the FoM, which allows a high resolution to be maintained while attaining a high detection accuracy. This is possible thanks to the optimization and interplay of several parameters, such as suitable choices of nanomaterials with high RI contrast and without high losses, the use of D‐shaped fiber substrates with a high penetration depth of the evanescent field, the integration of fiber sensors into an ad hoc developed microfluidic system that enables us to maintain stable thermal and mechanical conditions for the fiber, thus avoiding any cross‐sensitivity issue,^[^ ^6^ ^]^ injecting biofluids at controllable flow rates, thus avoiding any turbulence conditions,^[^ ^58^ ^]^ and the use of reliable processes and protocols for the surface functionalization and related biological tests.

In general, although optical fiber sensors have the capability to detect target molecules from small^[^ ^59^ ^]^ to larger sizes,^[^ ^60^ ^]^ their performance may vary greatly, since the sensitivity and LoD are also influenced by the nature of the molecules involved, and in particular by their affinity.^[^ ^61^ ^]^ As a consequence, any new assay involving a different target molecule has to be recalibrated, and different outcomes and performance may be found. However, it is possible to optimize the system and to tune it to work in the best conditions depending on the target molecule, while maintaining a very low LoD in the femto/attomolar range. Finally, it is also worth pointing out that the body fluid used (urine, blood plasma, serum, cerebrospinal fluid, etc.) can affect the kinetics of the binding interactions in different ways, due to the hydrophobic and electrostatic character of the molecules and proteins involved.^[^ ^62^ ^]^ Hence, once the body fluid has been selected as a consequence of a specific clinical need, the system must be calibrated, and eventually optimized, using the selected complex matrix.

## 3 Conclusion

In this paper, we have proposed and developed D‐shaped fiber sensors coated with stacks of TiO_2_ and Al_2_O_3_ nano‐layers, thus enabling the generation of BSW resonances. This approach is shown to yield superior performance compared to previous schemes using other metal oxides such as SnO_2_ and CuO. Our experimental results show that the proposed BSW‐based fiber sensors exhibited a five‐fold increase in RI sensitivity, mainly due to the increased RI contrast between the two nano‐assembled materials, which at the same time enabled the use of a reduced number of layers to achieve the bandgap behavior of the 1D photonic crystal. In addition, the resonance width of the BSWs was reduced compared to previous schemes, resulting in a 10‐fold improvement in the FoM on average. Both TE‐BSWs and TM‐BSWs demonstrated similar sensitivity but different resonance depths, and it is possible to use this degree of freedom further to select the most suitable BSW resonance for sensing purposes. In any case, the degrees of freedom of the proposed platform are quite numerous. For the sake of simplicity and to achieve good reproducibility in the manufacturing process, the thickness of the high and low RI layers was set to the same value, but the effects of modifying these thicknesses could be explored further. Another critical parameter is the losses of the materials. We selected two materials with the lowest possible extinction coefficient, as low losses typically generate optical resonances with a lower FWHM, and hence a better FoM. However, it is worth pointing out that in previous studies of LMRs, there has been an optimal value where the depth of the optical resonance is maximized with a good FWHM,^[^ ^25^, ^63^, ^64^ ^]^ and a similar effect has recently been studied in depth with the integration of an intermediate layer between the substrate and the nano‐film that generates the LMR.^[^ ^65^, ^66^ ^]^

One of the most interesting outcomes we would like to point out is that the FoM or sensitivity to RI alone can barely be used as a global parameter to really assess the performance of an optical device, while these parameters can just be used to preliminary envisage the performance as biosensor. In addition, we propose to use the reasonable figure of merit (RFoM) rather than the traditional FoM, in order to take also into account the depth/visibility of the optical resonance, an important parameter sometimes disregarded. Overall, the optical resonance‐based fiber sensor developed here opens up the possibility of using nanomaterials with even higher RI contrast and lower losses to achieve further optimization of sensor designs that may compete with and perhaps outperform the top technologies in the field.

Our assessment of the biosensing performance indicates an almost 15‐fold improvement in LoD compared to previous schemes in the literature where a single nano‐film was deposited onto D‐shaped fibers. To attain such extraordinarily low LoD in the attomolar range, an optimization of many other parameters was realized as discussed previously. IgG is used as a reliable biorecognition element for affinity‐related biological tests, due to its low cost and chemical stability. The target analyte was spiked in human serum to mimic a possible clinical scenario in which the measurement of IgGs can be applied when antibody deficiency (immunodeficiency) is suspected.^[^ ^67^ ^]^ Clearly, the proposed platform has the potential to be applied to the detection of any type of biomolecule, even targets with low molecular weight. In addition, researchers are putting a great deal of effort into the development of multi‐target analyte sensing systems based on multiple fiber‐based sensors, or, even more fascinatingly, by embedding this system in a single fiber through the exploitation of the differentiated local surface functionalization of the fiber sensing region. Finally, the integration of photonic systems with interdisciplinary fields such as biology and nanomaterials has enabled the quantification of substances for various applications in clinical, environmental, and industrial settings. This progress is paving the way for transitioning fiber‐based detection technology from the laboratory to the market.

## 4 Experimental Section

### Numerical Simulations

The band diagrams in Figure 2a,b were created using the transfer matrix method,^[^ ^55^ ^]^ while the transmission spectra were calculated using the finite difference method (FDM) and a finite element method (FEM) solver, based on the waveguide mode solver of FIMMWAVE software and the optical propagation tool FIMMPROP. More details of the mesh size and the parameters used for the FEM solver were given in the Supporting Information. The mesh can be observed in Figure S5 (Supporting Information), while the optical field intensity of the core for the TE‐BSW and the TM‐BSW modes were detailed in Figure S6 (Supporting Information). These profiles were calculated based on the central resonance wavelength of the structure achieved when the dispersion curves of the core‐guide mode and the surface modes intersected, as shown in Figure S7 (Supporting Information).

The methodology used for the simulations was the same as that described in ref. [31] where the silica RI of the fiber was calculated using the Sellmeier equation with the coefficients given in ref. [68] The complex value of the RI for the two materials used in the stack, i.e., TiO_2_ and Al_2_O_3_, was obtained based on measurements carried out with an ellipsometer, as described in the next section.

### Material Characterization and Imaging

**Figure 6:**
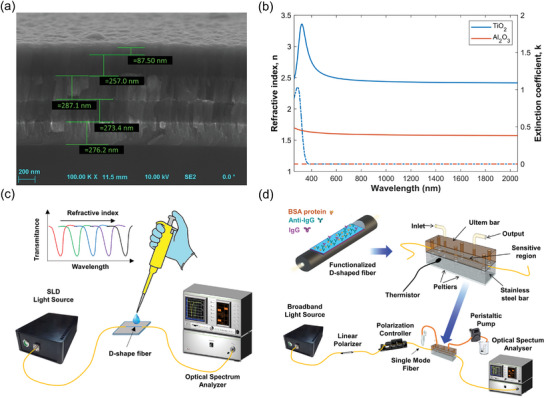
Optical characterization of the nanomaterials and experimental setups. a) Characteristic SEM image of the fiber cross‐section, highlighting the deposition of five nano‐films (TiO_2_, Al_2_O_3_, TiO_2_, Al_2_O_3_, TiO_2_) using the reactive magnetron sputtering technique. b) Optical characterization of the permittivity of the two nanomaterials (TiO_2_ in blue and Al_2_O_3_ in orange) in terms of refractive index, n (solid lines), and extinction coefficient, k (dashed lines). c) Experimental setup designed for the optical characterization of the fiber sensor in response to volume refractive index changes. d) Experimental setup configured for the detection of biological samples, wherein the fiber sensor is integrated into a microfluidic system, and solutions are injected through a peristaltic pump.

The D‐shaped fibers were standard single‐mode fibers, Corning SMF‐28 (cladding/core diameter of 125/8.2 µm), with a side‐polished length of 10 mm. These fibers were progressively polished down until an attenuation of 1 dB at 1550 nm was attained in RI‐matching oil (1.5 RIU). The polished surface of the optical fibers, henceforth called the sensitive region, was then coated with the five‐layer stack. In **Figure** **6**a, the stack of five layers were depicted, with values approximating the design of 260 nm for each layer, except for the last layer, which measures 87.5 nm (Figure S4, Supporting Information shows the same profile but with the deposition on a silicon wafer preform in parallel to the deposition on the D‐shaped fiber; the values were similar, with around 260 nm per layer except for the last layer of 74 nm). The thin film depositions were carried out using an Oxford Plasmalab 400 reactive magnetron sputtering system, supported with high‐purity 8‐inch Al and Ti targets. The additional Al_2_O_3_ coating, which was used to determine the sensitivity of the device to the formation of a biolayer, was deposited using the Veeco Savannah S100 atomic layer deposition setup, following the procedure described in ref. [69]

Reference TiO_2_ and Al_2_O_3_ coatings deposited on Si wafers were characterized using the Horiba Jobin‐Yvon UVISEL spectroscopic ellipsometer (as shown in Figure 6b), which provided great contrast between the materials over a broad wavelength range that included the operating range of the ellipsometer. In addition, it was note that the extinction coefficient was close to zero in the range 400–2000 nm, which includes the operating range for the experiment. However, although the ellipsometry results gave a negligible value, it was necessary to consider a non‐zero value in the numerical analysis in order to reach agreement with the experiments. The reason for this stemmed from the fact that the angle of incidence of the ellipsometer that was required to achieve reliable and accurate measurements, which was around 70°, did not provide precise results,^[^ ^68^ ^]^ and a value of 0.01 was chosen as this assured the best correlation between the numerical and experimental results. The deposition conditions were described in ref. [32]

### Experimental Setup

To carry out the experiments on the optical characterization of the devices in terms of volume RI changes, the D‐shaped fibers were connected at one end to a multi‐SLD light source (FJORD‐X3‐1330‐1650, Pyroistech) and at the other end to an optical spectrum analyzer (OSA) MS9740A from Anritsu (see Figure 6c). The fibers were also immersed in various solutions of glycerol in water at different concentrations: 0 (milli‐Q water), 10%, 20%, 30%, 40%, 50%, 60%, and 70% W/V, corresponding to an RI range of 1.333–1.429. The same process was followed after the deposition of a 64 nm coating of Al_2_O_3_ to mimic a biolayer on top of the five‐layer structure, and repeated once again after the etching of the Al_2_O_3_ layer by immersion in NaOH 0.1 M.

However, the experimental setup used for optical characterization of the devices in terms of surface RI changes was slightly different. The biosensing performance of the proposed device was assessed by building up a further setup (see Figure 6d) that consisted of a multi‐LED light source (Fibrelabs, Inc., SLD1310/1430/1550/1690), a polarization control system, a broadband OSA with a high optical resolution of 0.1 nm (Yokogawa, AQ6370D), a peristaltic pump (GILSON Minipulse 3) to inject the liquid solutions at controllable rates, and a computer connected via Ethernet to the OSA with a built‐in software program that was custom‐developed to record and process the optical signal with an improved detection accuracy and precision at the level of 10^−3^ nm (see Figure S8, Supporting Information). It was noted that the polarization control system was critical, as it allowed not only to separate the BSW polarization light components, but also to optimize the BSW optical features in order to achieve optical resonance that was as deep as possible. All the other details can be found in previous literature.^[^ ^16^ ^]^

### Chemical and Biological Reagents

All reagents, unless otherwise stated, were purchased from Merck (Milan, Italy). 1‐ethyl‐3‐(3‐(dimethylamino)‐propyl) carbodiimide hydrochloride (EDC), and N‐hydroxysuccinimide (NHS), mouse IgG and goat anti‐mouse‐IgG were purchased from Life Technologies Italia (Milan, Italy). Human serum (C Reactive Protein Free Serum) was purchased from HyTest Ltd. (Turku, Finland).

### Surface Functionalization of Nano‐Photonic D‐Shaped Fibers and Related Biosensing Response to Anti‐IgG Concentrations

The biosensing assessment was conducted through the development of model assays in which anti‐IgG was used as the target analyte. Any biosensing surface requires a suitable functionalization; in this case, the D‐shaped region of the fibers was nano‐functionalized with a thin layer (roughly 60 nm in thickness^[^ ^70^ ^]^) of Eudragit L100 (Evonik) copolymer that provided the free functional groups necessary for antibody grafting onto the sensor surface. To reach this goal, the sensitive region of the fiber was immersed in 2 mM (0.04% w/v dissolved in ethanol) Eudragit L100 for 1 min, and was left to dry in air for 15 min. Following this, the sensing region of the fiber was glued with a suitable UV‐curing optical adhesive (NOA 68, Norland Products, Inc.) inside the microfluidic system, to avoid any mechanical effect or fiber bending, while the rest of the fiber was fixed to the optical bench with “magic” tape to avoid any effects due to polarization changes of light. The microfluidic system allowed us to handle small liquid solutions on the order of tens of µL and to keep the temperature of the device stable during the long‐term experiments, with maximum oscillations of ±0.1 °C and σ < 0.05 °C thanks to the use of an ILX Lightwave LDC‐3722B TEC controller in feedback loop mode, which can guarantee a thermal accuracy up to ±0.01 °C.

In the next steps, the temperature was fixed at 22 °C (±0.05 °C), and the functional groups were activated by a solution of EDC/NHS (2 mM/5 mM, respectively), which was injected for less than 20 min at a flow rate of 25 µL min^−1^. This time was considered sufficient to guarantee proper activation of the functionalities, which occurs in less than 15 min. To achieve covalent grafting of the suitable BRE onto the sensing fiber region, 500 µg mL^−1^ of IgG antibody in PBS solution was then immediately injected for 1 h at a flow rate of roughly 15 µL min^−1^, and this was followed by rinsing in PBS for 10 min (until a stable level of the optical signal λ_BSW_ was reached) at a flow rate of 100 µL min^−1^ in order to remove unreacted antibodies. To conclude the preparation of the biosensing surface, a solution of BSA (0.1% w/v) was first injected for 25 min at a flow rate of 25 µL min^−1^ to block the remaining active groups on the sensing surface (blocking agent function) and to prevent the non‐specific adsorption of biomolecules (surface passivation); another rinsing in PBS was then applied to reach a stable signal. The value of the optical signal λ_BSW_ at this point defined the starting value for the baseline in the biosensor response to subsequent analyte detection.

Before serially injecting the specific analyte, the biosensor specificity was evaluated by injecting a solution of 1:10 PBS‐diluted human serum as a negative control for 20 min (at least until a stable level of the optical signal λ_BSW_ was reached) at a flow rate of 20 µL min^−1^. The assay was then finalized by detecting a wide range of increasing concentrations of anti‐IgG ranging from 100 fg mL^−1^ to 10 µg mL^−1^ (six concentrations plus the blank sample) and spiked in human serum, following the same detection protocol: analyte injection for 20–25 min at a flow rate of 20 µL min^−1^, subsequent rinsing in PBS for 10 min (at least until a stable level of the optical signal λ_BSW_ was reached) at a flow rate of 100 µL min^−1^. The measurement window at flow was then stopped for 5–10 min, and roughly 25 experimental points were recorded (to avoid any influence/interference due to temperature fluctuations, flow variation, and solution bulk effect). It was worth highlighting that since the device was sensitive to different RI values of the solutions (volume RI change), rinsing in PBS plays a vital dual role, as it removes the excess analytes and enables measuring the real shift of the optical signal λ_BSW_ at each step caused by the antibody–analyte binding interaction.^[^ ^6^ ^]^

### Statistical Analysis

Data were normalized at the pre‐processing stage and then expressed as a mean ± standard deviation after the elaboration. Three independent repetitions (*n* = 3) of each experiment were considered, and a dataset of 30 samples was created for each experimental point. The Origin 2022 software program and integrated functionalities were used to perform the statistical analysis and prepare the related graphs.

## Conflict of Interest

The authors declare no conflict of interest.

## Author Contributions

I.D.V., F.C., P.T., and M.S. conceived the idea. I.D.V., M.S., P.T., I.M., and F.C. supervised the project, E.G.V., N.K., and M.J. analyzed the data., E.G.V. performed the numerical analysis. N.K., D.B., D.A., E.P., and A.G. fabricated the materials and carried out experiments. All authors contributed to the preparation of the manuscript. I.D.V., N.K., and, E.G.V. equally contributed to this work.

## Supporting information

Supporting Information

## Data Availability

The data that support the findings of this study are available from the corresponding author upon reasonable request.
