# Supplementary material for: Nano‐Photonic Crystal D‐Shaped Fiber Devices for Label‐Free Biosensing at the Attomolar Limit of Detection
Source: Adv Sci (Weinh). 2024 Jul 23;11(35):2310118. doi: 10.1002/advs.202310118 (PMC11425293; doi:10.1002/advs.202310118)
Supplement: Supplementary file 1 — Supporting Information [file ADVS-11-2310118-s001.pdf]

Supporting Information

Nano-Photonic Crystal D-shaped Fiber Devices for Label-Free Biosensing at the Attomolar Limit of Detection

Ignacio Del Villar, Esteban Gonzalez-Valencia, Norbert Kwietniewski, Dariusz Burnat, Dayron Armas, Emil Pituła, Monika Janik, Ignacio R. Matías, Ambra Giannetti, Pedro Torres, Francesco Chiavaioli,* and Mateusz Śmietana**

I. Del Villar, D. Armas, I. Matías
Electrical, Electronic and Communications Engineering Department, Public University of Navarre, 31006 Pamplona, Spain
ignacio.delvillar@unavarra.es

I. Del Villar, I. Matías
Institute of Smart Cities (ISC), Public University of Navarra, 31006 Pamplona, Spain
ignacio.delvillar@unavarra.es

E. Gonzalez-Valencia
Department of Electronic and Telecommunications Engineering, Instituto Tecnológico Metropolitano, 050013, Medellín, Colombia.

E. Gonzalez-Valencia, P.Torres
Escuela de Física, Universidad Nacional de Colombia - Sede Medellín, A.A. 3840, 050034, Medellín, Colombia.

N. Kwietniewski, D. Burnat, E. Pituła, M. Janik, Mateusz Śmietana
Warsaw University of Technology, Institute of Microelectronics and Optoelectronics, 00-662, Warszawa, Poland
mateusz.smietana@pw.edu.pl

A. Giannetti, F. Chiavaioli

National Research Council of Italy (CNR), Institute of Applied Physics “Nello Carrara”, 50019 Sesto Fiorentino, Italy
f.chiavaioli@ifac.cnr.it

Photonic bandgap analysis

The thickness of the photonic crystal layered structure was chosen by calculating the dispersion band diagram for the semi-infinite multilayer using the transfer matrix method[1]. The β-axis represents the propagation constant of the incident light, which is parallel to the surface of the multilayer structure, and the frequency axis indicates the wavelength range of interest[2]. Given that the materials for the thin film depositions had already been defined, the possible combinations of film thicknesses from 50 to 500 nm were analyzed for both materials. **Table S1** summarizes the band diagram of the developed 1DPC for some of the Al_2O_3 and TiO_2 thicknesses. A photonic bandgap is seen for both polarizations in the wavelength region of interest, where the yellow and red dots represent the BSW resonances when the fiber is immersed in an aqueous medium. The figures show that for the thickness combinations studied here, the band diagrams have very small variations, thus confirming the selection of the optimized thickness for the 260/260 nm stack. In addition, the TM bandgap of interest is slightly wider for thicker TiO_2 layers; however, this would complicate the deposition process, since the total thickness of the 1DPC would be too high.

Table S1. Dispersion band diagram for different thicknesses of the 1DPC layered structure.

Layer thickness	TE Band diagram	TM Band diagram
100 nm for Al_2O_3 and 310 nm for TiO_2	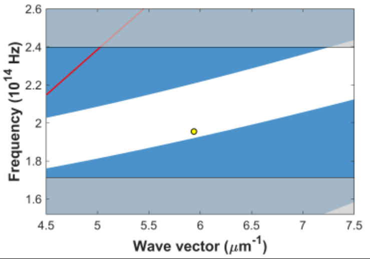	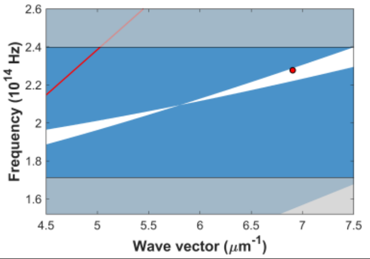

150 nm for Al_2O_3 and 290 nm for TiO_2	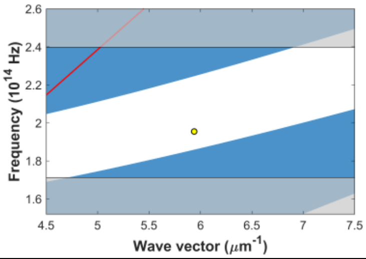	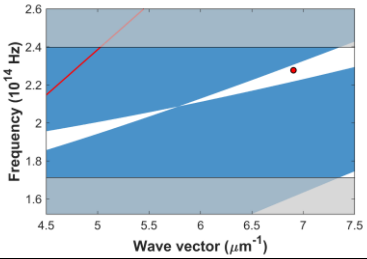
200 nm for Al_2O_3 and 280 nm for TiO_2	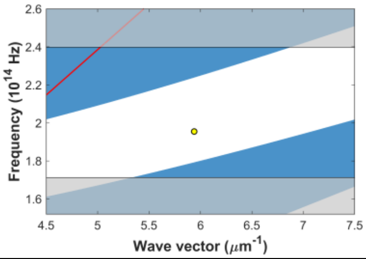	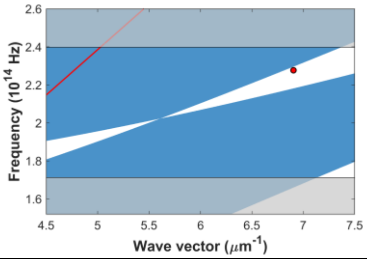
250 nm for Al_2O_3 and 260 nm for TiO_2	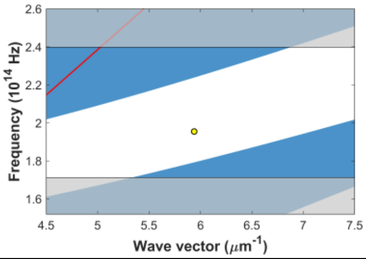	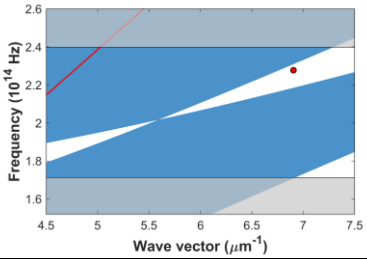
300 nm for Al_2O_3 and 250 nm for TiO_2	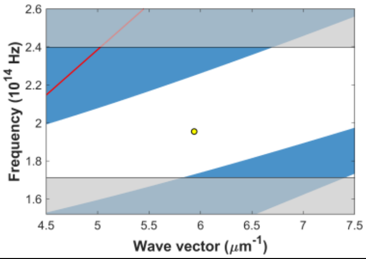	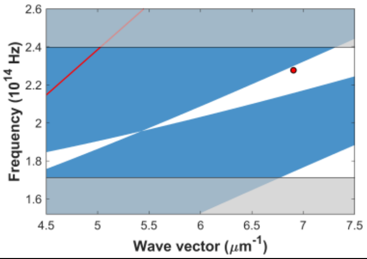
350 nm for Al_2O_3 and 230 nm for TiO_2	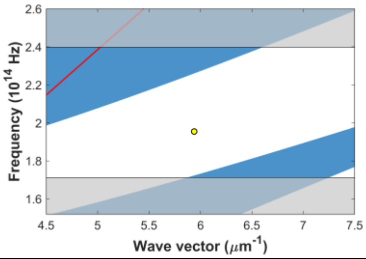	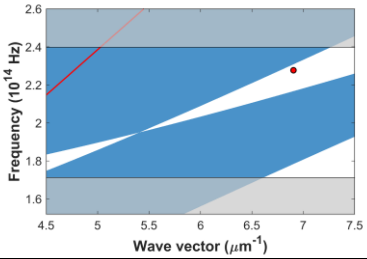
400 nm for Al_2O_3 and 220 nm for TiO_2	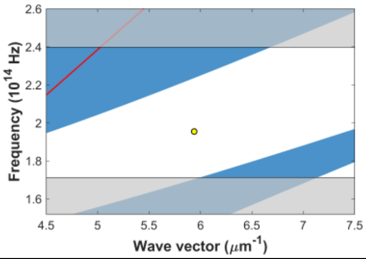	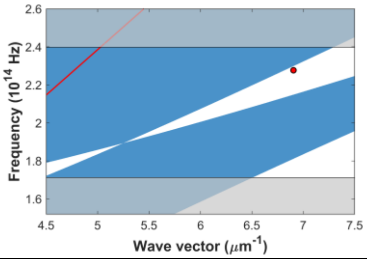

450 nm for Al_2O_3 and 210 nm for TiO_2	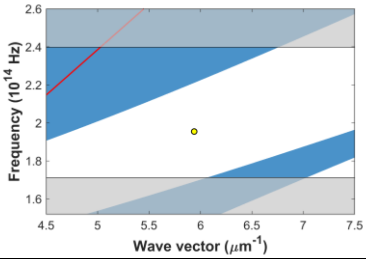	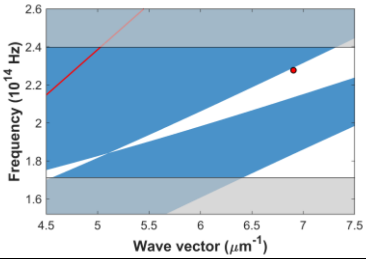
500 nm for Al_2O_3 and 190 nm for TiO_2	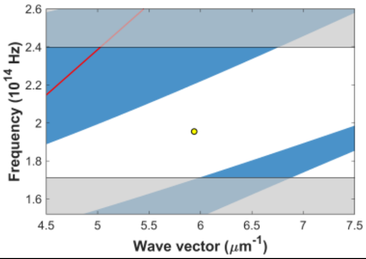	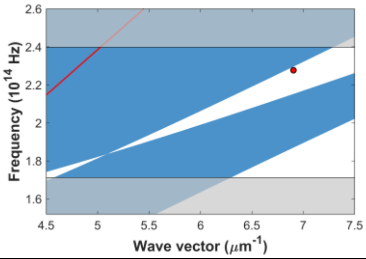

Evaluation of the sensor performance with an Al_2O_3 biological mimicking biolayer

To evaluate the performance of the five-layer stack deposited on a D-shaped fiber as a biosensor, Fiber 2 (with a final TiO_2 layer of 74 nm) was chosen and an additional film of Al_2O_3 was deposited on it, as this material has a refractive index (RI) close to those of some biofilms detected by biosensors ($n_{biofilm}$ =1.55–1.6 according to ref. [3]). **Figure S1** shows the transmission spectra for different surrounding medium RIs, which enable us to assess the sensitivity of the system. The TE-BSW is located at longer wavelengths due to the increase in the thickness of the Al_2O_3 layer deposited on the D-shaped fiber, as observed in other works[2,4]. However, this wavelength shift does not lead to a higher RI sensitivity. **Figure S2** illustrates the resonant wavelength shift with and without the additional layer, and we can see from this graph that the sensitivity of Fiber 2, initially 1281 nm/RIU, decreases to 835 nm/RIU after the deposition of the Al_2O_3 layer. This reduction with a further low RI layer is as expected, since the additional layer leads to a reduction in the penetration depth of the wave-associated evanescent field in the external medium compared to Fiber 2 without the additional layer. This information is given in **Table 1** in the main document, along with the FWHM and FoM (with values of 9.5 nm and 87.9 RIU^{-1}, respectively higher and lower than without the biolayer).

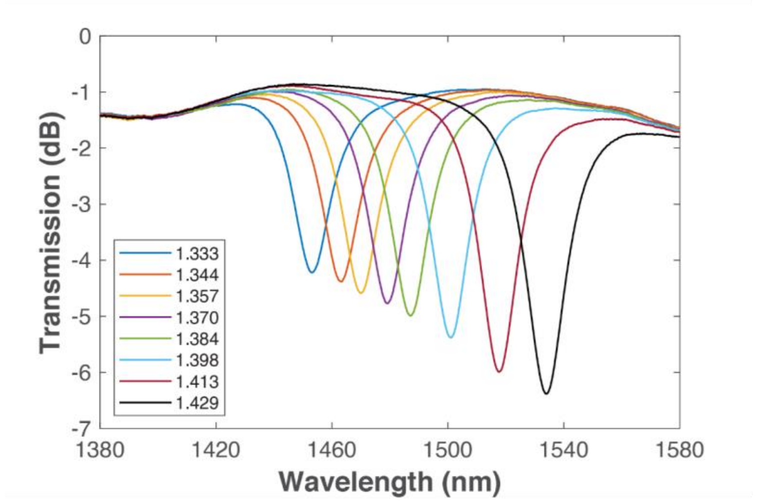

Figure S1. Evolution of the transmission spectrum with a biological mimicking layer. Transmission spectra are shown for refractive indices in the range 1.333–1.429 for Fiber 2 with an additional Al_2O_3 layer.

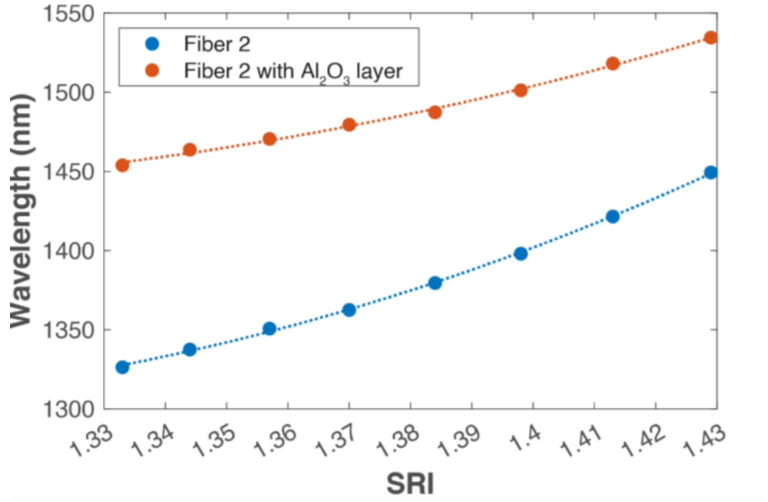

Figure S2. Sensitivity comparison of Fiber 2 with and without the biological mimicking layer**.** The wavelength shift in the BSW resonance achieved from Fiber 2 is shown as a function of the refractive index with and without the Al_2O_3 layer.

After deposition, the device was subjected to an etching process by immersing it in NaOH 0.1 M according to the process used in ref. [5]. **Figure S3a** shows the progressive spectral shift of the BSW to shorter wavelengths during the etching process, while **Figure S3b** shows the wavelength evolution of the BSW minimum value as a function of time. After a very fast and sharp change during the first 30 s, the variation becomes slower and finally stabilizes after 150 s, indicating that the Al_2O_3 layer has been completely removed. The wavelength of the original

BSW resonance was 1330 nm before the deposition of the Al_2O_3 layer. In addition, the rate of removal was 0.4 nm/s, as the Al_2O_3 layer was approximately 60 nm thick.

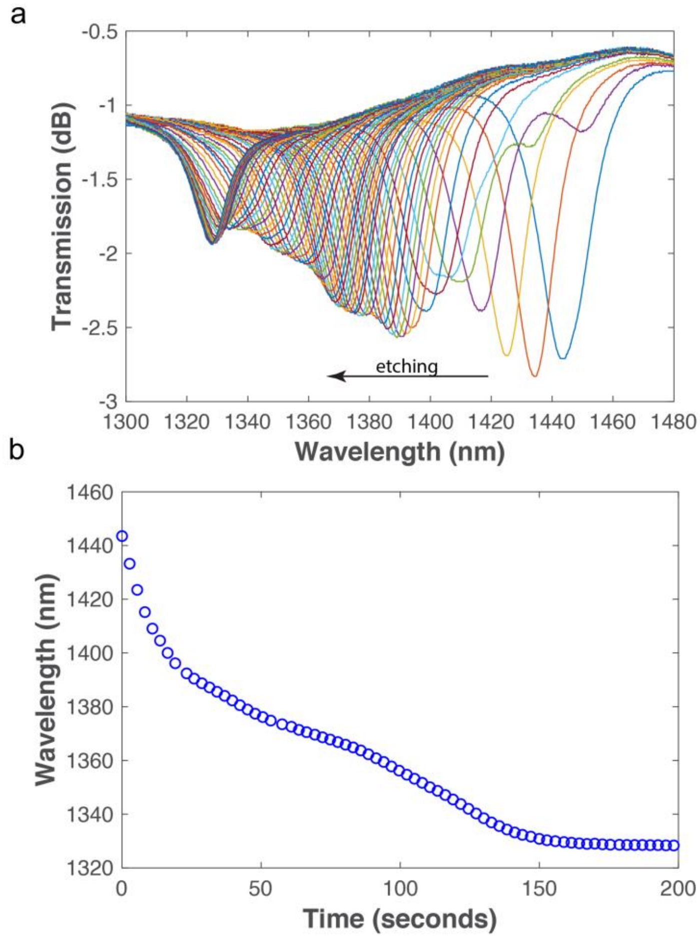

Figure S3. Spectral analysis of the biological mimicking layer during the etching process. **(a)** Transmission spectra monitored during the etching process of Al_2O_3 on a D-shaped fiber using Fiber 2. **(b)** Evolution in the wavelength shift of BSW resonance during the etching process.

In contrast, the TiO_2 layer was not affected under these experimental conditions, as it is significantly more chemically resistant material than Al_2O_3, and is generally removed using strong acids, such as hydrofluoric acid (HF) or phosphoric acid (H_3PO_4) [6,7]. This is also the reason why TiO_2 was selected as the last layer, since it is suitable for both testing the sensitivity and for the addition of the biological mimicking layer, which can be deposited and then easily removed. Following this, the device can be used for the final biosensing application. Moreover, since the removal of the biological mimicking layer is not immediate, the process could be stopped several times to analyze the sensitivity at different thicknesses, thus imitating the different steps of the binding interaction in a biosensor.

Characterization of the stack of thin films via imaging

Figure 6a in the main document shows a SEM image of the stack of five layers deposited on Fiber 2, which made it possible to attain values approaching the optimal design in which each layer has a thickness of 260 nm and the last layer a thickness of 87.5 nm. In parallel, in order to assess the reliability and repeatability of deposition, the same structure deposited on the D-shaped fibers was manufactured on a silicon wafer. A SEM image of the cross-section of the wafer is shown in **Figure S4**, where values comparable with the error in the deposition technique (±15 nm) can be observed: around 260 nm for the first four layers, and 74 nm for the last layer (this value was slightly different from the 87.5 nm measured for the optical fiber, due to the accuracy of the SEM image and the fact that the structure was deposited on a different substrate). This finding confirms that silicon, which is much easier to cut and handle than D-shaped fiber, can be used as a reference to monitor the thickness of the different layers in the deposition process. Moreover, this means that the need to destroy the optical fiber sample can be avoided, which should not be underestimated.

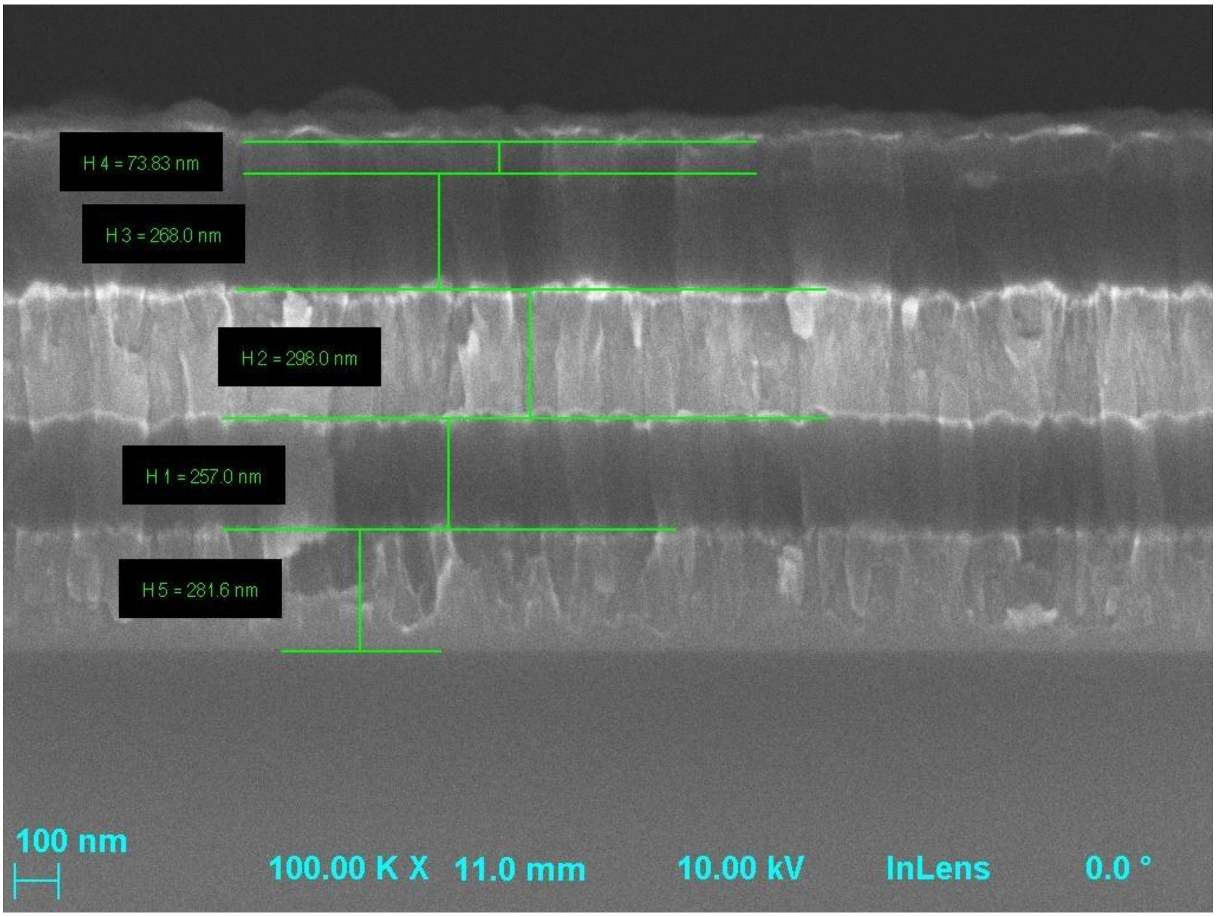

Figure S4. Characteristic SEM image of the stack of thin films in silicon wafer. Zoomed cross-sectional SEM image of the designed stack of five nano-films (TiO_2, Al_2O_3, TiO_2, Al_2O_3, TiO_2) deposited by a reactive magnetron sputtering technique on a silicon wafer for comparison purposes.

Numerical analysis using FIMMWAVE software

FIMMWAVE® was used to analyze the transmission through the five-layer stack coated on the D-shaped fiber. The propagation analysis and outcomes were obtained with FIMMPROP, a module integrated with FIMMWAVE. Three sections were defined: a standard SMF segment, a coated D-shaped SMF segment, and another standard SMF segment. All three sections were studied using the FOM Solver, based on the finite element method (FEM). The overall density of the FEM mesh was defined and controlled by the parameter *nElemPerDiagonal* = 100, which set the number of triangles along the diagonal of the waveguide, while the additional parameters for the FEM-based solver were: *smallestFeatureFrac* = 0.001, *anglePriority* = 0.5, *minAngle* = 30º and *edgeRefineCoeff* = 0. The meshes established for the standard SMF and the coated D-

shaped SMF were set as shown in **Figure S5**, where it can be seen that only half of the structure was numerically analyzed due to its symmetry.

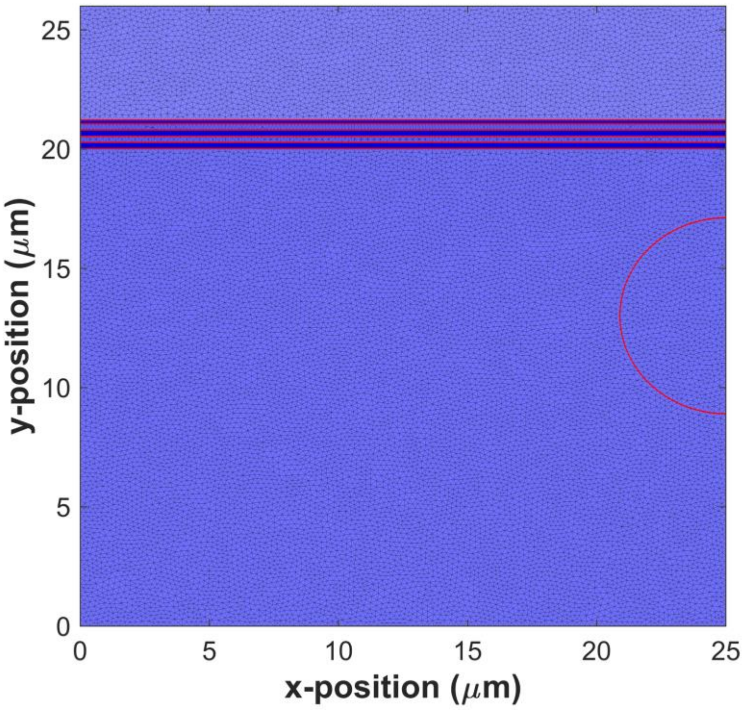

Figure S5. Definition of mesh parameters in FIMMWAVE software. Mesh used in FIMMWAVE for the FEM Solver for the D-shaped SMF segment.

The standard SMF section allowed us to calculate the single mode that was guided into the core of the D-shaped section. This guided mode was the one whose variation due to coupling to other modes was taken into account in order to obtain the transmission spectrum when the RI of the surrounding medium was lower than that of the optical fiber. No phase-matching layer (PML) was used, in order to simplify the computational process and to dramatically reduce the computational cost and time. The optical distributions of the core-guide mode and the surface modes (TE-BSW at TE polarization and TM-BSW at TM polarization) are shown in **Figure S6**.

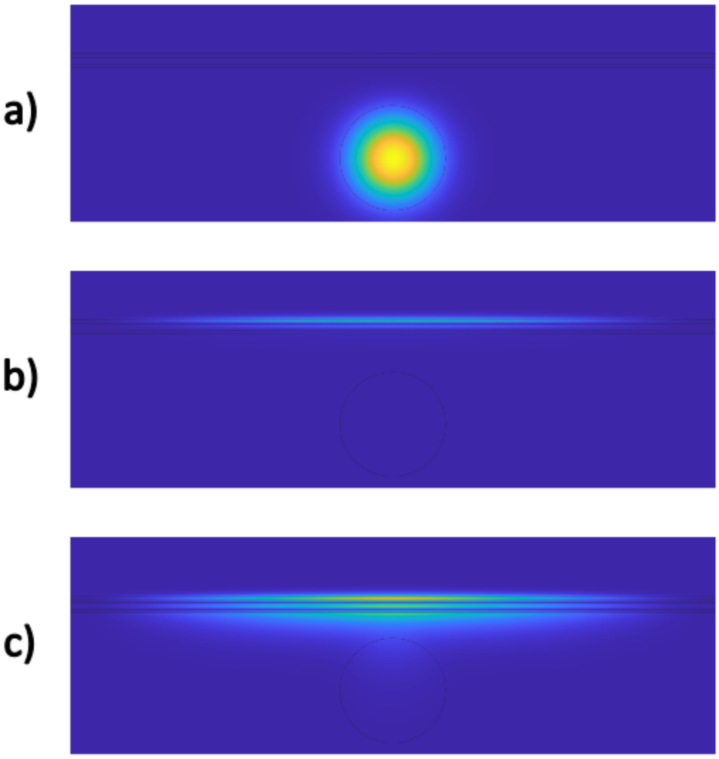

Figure S6. Optical field intensity distributions for different modes: **(a)** core-guided mode, **(b)** TE-BSW, and **(c)** TM-BSW.

The dispersion curves for the core-guide mode and the surface modes cut at the resonant wavelength were also studied in depth. At this wavelength, the light was phase-matching coupled from the fiber core to the five-layer stack, and a dip or attenuation band in the transmission spectrum was generated. **Figure S7** shows the transmission spectra and dispersion curves for both the TE and TM polarization states of light.

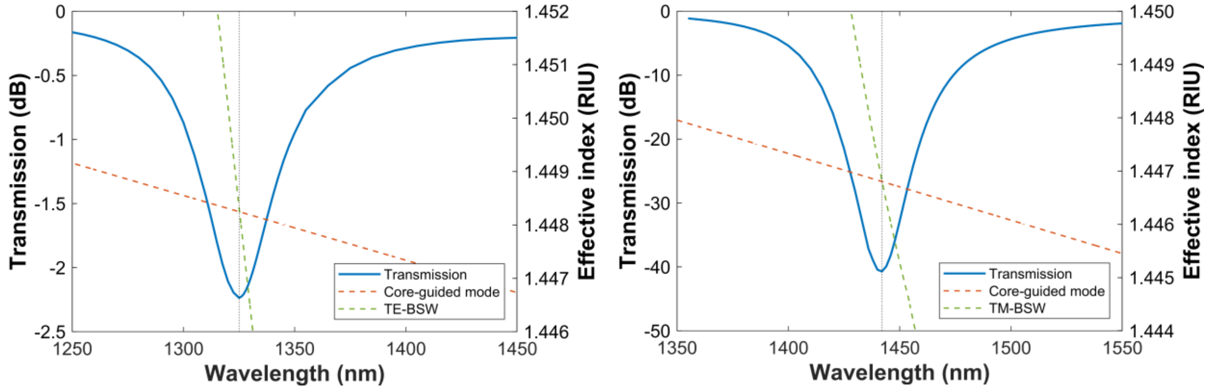

(a) **(b)**

Figure S7. Transmission spectra and dispersion curves for both the core-guided mode and surface modes for **(a)** TE and **(b)** TM polarization.

Software interface for data recording and processing

As discussed in previous work[8], when the aim is to accurately monitor the spectral features of optical resonance (for instance, the minimum or maximum values of the wavelength or amplitude) generated by any physical phenomenon, it is necessary to use *ad hoc* developed software to carry out data recording and processing in order to improve the detection resolution and, ultimately, the limit of detection in the case of a biosensor[9]. To reduce the error (and hence the standard deviation) in the detection of the minimum wavelength (λ_{BSW}) value, a home-made software routine was developed, and a picture of the software control panel is shown in **Figure S8**. The steps in the software routine are summed up here below:

1. The OSA span (or bandwidth) is selected to match the effective bandwidth of the optical resonance, with the central wavelength roughly corresponding to the expected value of λ_{BSW}, and this span determines the spectral resolution for a fixed value of the number of samples (in our case, the span was set to 150 nm to observe the entire BSW resonance, as shown in the top chart in **Figure S8,** giving a spectral resolution of $\frac{150\,nm}{7500\,samples} = 0.02\,nm$).
2. The effective number of points to which the fitting function is applied can be inserted in the box marked "subarray for fit". This enables us to select only a portion of the entire optical resonance, and hence determines the effective bandwidth of the fitting process (in our case, the effective bandwidth was $0.02\,nm \cdot 700 = 14\,nm$, since the subarray for the fit was 700), and the effective accuracy, precision and resolution of the assessment of λ_{BSW}.
3. The fitting function can be selected via the box marked "fitting function." It is possible to use different types of fitting function, including linear (polynomial of different orders) and nonlinear functions (Gaussian and Lorentzian), depending on the shape that best suits the optical resonance.
4. When a nonlinear fitting function is chosen, the starting value of the minimum wavelength (λ_{BSW}) must be inserted in order to avoid errors in the fitting process.
5. The acquisition time for each experimental point during continuous measurement can be set in the right-top box, marked "acquisition period," which enables us to monitor the kinetics of interaction in real time during the binding events among biomolecules.
6. The right-bottom box, marked "mean square error," provides the error in the calculation

of the λ_{BSW} for each acquisition (in our case, the error ranges from 10^{-3} to 10^{-2} nm at the maximum).

7. The software outputs a file that can easily be imported into data analysis software (Excel, Origin, etc.) and consists of three columns: time, λ_{BSW}, and the error in the estimation of λ_{BSW}.

It is clear that if the fitting process is carried out based on the FWHM of the optical resonance (roughly 40 nm), the error in the determination of the λ_{BSW} will be greater than it actually is.

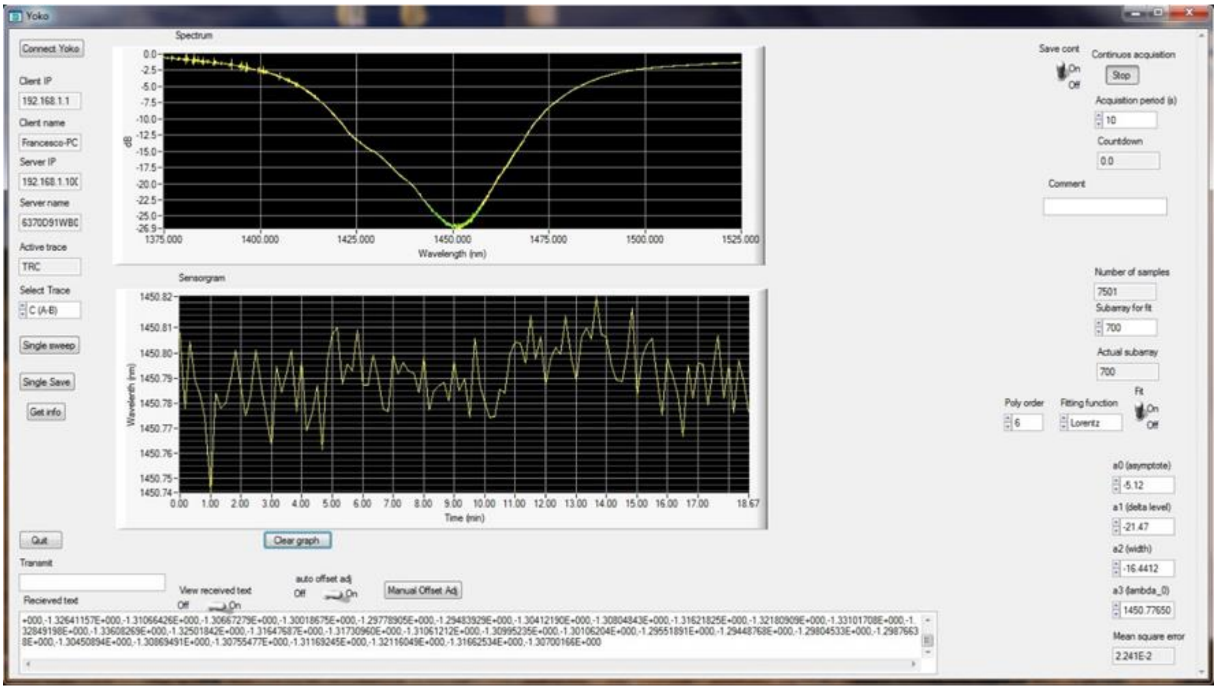

Figure S8. Software control panel. The developed software shows on the top chart the fiber transmission spectrum centered at the minimum of λ_{BSW}. The yellow curve is the raw spectrum, whereas the green curve is the fitting curve generated from the raw data. The bottom chart details the real-time evolution of the fitted value of λ_{BSW}.

References

[1] P. Yeh, A. Yariv, C.-S. Hong, *J Opt Soc Am* **1977**, *67*, 423.

[2] E. Gonzalez-Valencia, I. Del Villar, P. Torres, *Sci Rep* **2021**, *11*, 11266.

[3] S. Bandyopadhyay, I. Del Villar, N. Basumallick, P. Biswas, T. K. Dey, S. Bandyopdhayay, *Journal of Lightwave Technology* **2017**, DOI 10.1109/JLT.2017.2754549.

[4] F. J. Arregui, I. Del Villar, C. R. Zamarreño, P. Zubiate, I. R. Matias, *Sens Actuators B Chem* **2016**, *232*, DOI 10.1016/j.snb.2016.04.015.

[5] M. Śmietana, M. Janik, M. Koba, W. J. Bock, *Opt Express* **2017**, *25*, 26118.

[6] S. Kondati Natarajan, A. M. Cano, J. L. Partridge, S. M. George, S. D. Elliott, *The Journal of Physical Chemistry C* **2021**, *125*, 25589.

[7] S. Okazaki, T. Ohhashi, S. Nakao, Y. Hirose, T. Hitosugi, T. Hasegawa, *Jpn J Appl Phys* **2013**, *52*, 098002.

[8] F. Chiavaioli, P. Biswas, C. Trono, S. Jana, S. Bandyopadhyay, N. Basumallick, A. Giannetti, S. Tombelli, S. Bera, A. Mallick, *Anal Chem* **2015**, *87*, 12024.

[9] F. Chiavaioli, C. A. J. Gouveia, P. A. S. Jorge, F. Baldini, *Biosensors (Basel)* **2017**, *7*, 23.
